# Alkali-treated titanium dioxide promotes formation of proteoglycan layer and altered calcification and immunotolerance capacity in bone marrow stem cell

**DOI:** 10.1016/j.bbrep.2023.101569

**Published:** 2023-11-09

**Authors:** Tomomi Mizutani, Shuhei Tsuchiya, Masaki Honda, Jorge Luis Montenegro Raudales, Kensuke Kuroda, Hironori Miyamoto, Tomohisa Nakamura, Kenichiro Ishibashi, Yasuyuki Shibuya

**Affiliations:** a Department of Oral Maxillofacial Surgery, Nagoya City University Graduate School of Medical Sciences, 1 Kawasumi Mizuho-cho Mizuho-ku, Nagoya, Aichi, 467-8602, Japan; b Department of Oral Anatomy, School of Dentistry, Aichi Gakuin University, 1-100 Kusumoto-cho, Chikusa-ku, Nagoya, Aichi, 470-0131, Japan; c EcoTopia Science Institute, Nagoya University, Furo-cho, Chikusa-ku, Nagoya, Aichi, 464-8603, Japan

**Keywords:** Alkaline treatment, Titanium dioxide, Proteoglycan, Immunotolerance, T-cell, Macrophage, Bone marrow stem cells

## Abstract

**Introduction:**

In this study, we report that a proteoglycans (PGs)-layer between the bone and titanium dioxide (TiO_2_) surface after osseointegration improved the calcification capacity and immunotolerance of human bone marrow mesenchymal stem cells (hBMSCs) on TiO_2_. Alkaline treatment of TiO_2_ is a method for promoting osteogenesis in hBMSCs. We hypothesized that promotion of osteogenesis due to alkaline treatment was caused by changing PGs-layer on TiO_2_.

**Objective:**

This study aimed to analyze whether alkaline treatment of TiO_2_ affects PGs-layer formation and immunotolerance in hBMSCs.

**Methods:**

The topology and wettability of the alkaline-treated titanium (Ti-Al) and unprocessed titanium (Ti-MS) surfaces were characterized. Initial cell attachment, cell proliferation, calcification capacity, alkaline phosphatase activity, PGs-layer formation, PGs function, and the expression of osteogenic and immunotolerance-related genes were analyzed. The conditioned medium (CM) from hBMSCs grown on Ti-Al and Ti-MS was added to macrophages (hMps) and Jurkat cells, and immunotolerance gene expression in these cells was analyzed.

**Results:**

hBMSCs cultured on Ti-Al showed increased initial cell attachment, cell proliferation, PG-layer formation, and osteogenic capacity compared with hBMSCs on Ti-MS. Gene expression of indoleamine 2,3-dioxygenase (IDO) in the hBMSCs cultured on Ti-Al was higher than that in the hBMSCs on Ti-MS. CM from hBMSCs did not affect markers of M1 and M2 macrophages in hMps. CM from hBMSCs cultured on Ti-Al altered the gene expression of Foxp3 in Jurkat cells compared to that of CM from hBMSCs on Ti-MS.

**Significance:**

These results suggest that alkaline treatment of TiO_2_ altered PGs-layer formation, and changed the osteogenesis and immunotolerance of hBMSCs.

## Highlights

• BMSCs on alkali-treated TiO_2_ promoted expression of glycosyltransferases.
• BMSCs on alkali-treated TiO_2_ changed morphology of PGs-layer.
• Proteoglycans-layer on alkali-treated TiO_2_ promoted calcification capacity.
• BMSCs on alkali-treated TiO_2_ altered immunotolerance of macrophage and T-cells.

## Graphical abstract

**Figure:**
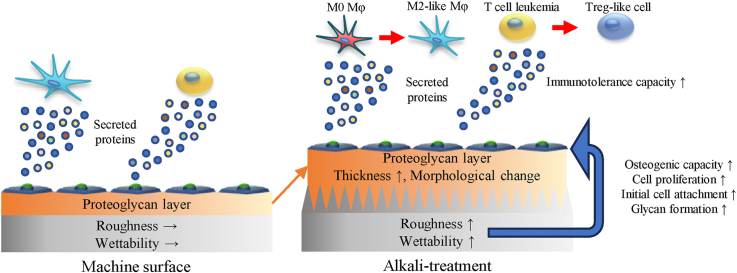

## 1 Introduction

Bone-marrow-derived mesenchymal stem cells (BMSCs) are multipotent stem cells that colonize the TiO_2_ surface after implantation and are associated with osteogenic capacity [1]. Several studies have discussed the interactions of BMSCs with TiO_2_ and have demonstrated osteogenic properties, cell adhesion, proliferation, and differentiation [[2], [3], [4], [5]].

A dental implant is an artificial root used in dentistry to support a prosthetic restoration with the goal of replacing missing teeth. Dental implant treatment success requires osseointegration, which involves direct contact between the bone and dental implant. This phenomenon occurs when BMSCs adhere and differentiate into osteoblasts on the implant surface. Titanium dioxide (TiO_2_) has been widely utilized for dental implants due to its positive influence on the wound healing process by virtue of its biocompatibility and mechanical strength [6], in addition to being an abundant natural resource. Various modifications to TiO_2_ surface have been tried to improve osseointegration [2,7], because the rate of success is about 95% [8] but not 100%.

Alkaline treatment of the TiO_2_ surface is simple and economical, and the nanotube dimensions can be precisely controlled compared with other treatments to improve osseointegration. Alkaline treatment changes the topology of the TiO_2_ surface into a micro/nanostructure and improves its wettability [9], which improve the attachment, proliferation, and osteogenic differentiation in BMSCs [1,[10], [11], [12], [13], [14], [15], [16], [17], [18]], even bone-induction capacity *in vivo* and *in vitro* [2,[19], [20], [21]]. Alkali-treated TiO_2_ affects its biocompatibility by controlling macrophage differentiation into the M2 phenotype, which modulates the inflammatory response [[22], [23], [24]]. However, the mechanisms of osteogenic capacity and biocompatibility of alkali-treated TiO_2_ and the molecular interactions that occur at the implant interface are yet to be fully elucidated.

Proteoglycans (PGs) and glycosaminoglycans (GAGs) are molecules abundant on the cell surface and in the extracellular matrix where they play pivotal roles in facilitating signal transduction and maintaining stem cell homeostasis [25], and also serve as key regulators of immune cell (leucocyte) recruitment and positioning during the inflammatory process [26]. PGs modulate osteogenic differentiation of MSCs [27]. 50–500 nm of PGs was observed between the bone and TiO_2_ surface, and this layer contained PGs without collagen fibers as the extracellular matrix (ECM) after osseointegration, as observed by transmission electron microscopy (TEM) [28]. PGs are heavily glycosylated by chondroitin sulfate (CS), dermatan sulfate (DS), keratin sulfate, and hyaluronic acid. These polysaccharides, consisting of repeating disaccharide units, are called glycosaminoglycans (GAGs), and are catalyzed by glycosyltransferases. PGs and GAGs are abundant on the cell surface and in the extracellular matrix where they play pivotal roles in facilitating signal transduction and maintaining stem cell homeostasis [29]. PGs modulate MSC osteogenic differentiation of mesenchymal stem cells [30]. The sulfation contained in PGs affects the osteogenic differentiation of stem cells because sulfate groups provide binding sites for multiple growth factors [31].

A previous report suggested that chondroitin-4-sulfate (C4S), a GAG, adsorbed on TiO_2_ strongly, compared to other glycans [32]. The role of the PG-layer on TiO_2_ is biological interfacial adhesion between the bone and titanium surface [33], but it does not alter the intrinsic strength of the mineralized tissue [34]. C4S transferase-1 (C4ST-1) catalyzes 4-O-sulfation in CS [35] and the PG-layer between hBMSCs and TiO_2_ is altered by C4ST-1, directly and indirectly affecting the immunotolerance capacity but not the osteogenic properties of hBMSCs on TiO_2_ [36]. Thus, the role of the PG-layer on TiO_2_ has not yet been fully elucidated.

We hypothesized that the alkali treatment of TiO_2_ affects the morphology and function of the PG-layer derived from hBMSCs. To test this hypothesis, we analyzed the gene expression of glycosyltransferases and the calcification capacity of PGs in BMSCs cultured on alkali-treated TiO_2_ *in vitro*. Furthermore, we analyzed the immunotolerance of BMSCs, T-cell, and human macrophages (hMps) on alkali treated-TiO_2_.

## 2 Materials and methods

### 2.1 Specimen preparation

Ti disks (80, 32, 15, and 6 mm in diameter and 2 mm in thickness) of grade 2 commercially pure Ti were prepared by machining (Ofa Co., LTD, Chiba, Japan). After polishing with #800 grinder, Ti O_2_ disks were coated with 4.0 mM HNO_3_ and 0.8 mM HF, then washed ultrasonically in 1% (w/v) sodium dodecyl sulfate (SDS) (FUJIFILM-WAKO, Osaka, Japan), acetone (FUJIFILM-WAKO), and 70% ethanol, for subsequent use. These TiO_2_ disks were immersed and treated with an ultrasonicator in 5.0 M NaOH aqueous solution and placed in bath maintained at 37 °C for 1 h (Ti-Al). Unprocessed TiO_2_ disks were used as controls (Ti-MS).

### 2.2 Surface characterization

The topographies of Ti-MS and Ti-Al were observed using high-resolution scanning electron microscopy (SEM) (S-4800; Hitachi, Tokyo, Japan). Wavelength dispersive spectrometry (WDS) was performed to evaluate the chemical composition of the sample surface. Surface wettability was evaluated using a contact angle measurement system (VSA 2500 XE; AST Products, Billerica, MA, USA).

### 2.3 Cell culture

The human BMSC cell line UE7T-13 was obtained from the Japanese Collection of Research Biosources (JCRB; Osaka, Japan). The cells were cultured in DMEM containing 10% fetal bovine serum (FBS) (Sigma-Aldrich, Tokyo, Japan) and 1% penicillin and streptomycin (PS) (FUJIFILM-WAKO) at 37 °C and 5% CO_2_ under humidified conditions. THP-1 monocytes and Jurkat cells were purchased from JCRB and cultured in RPMI 1640 medium containing 10% FBS and 1% PS. THP-1 monocytes were differentiated into human macrophages (hMps) by 24 h incubation with 150 nM phorbol 12-myristate 13-acetate (Sigma-Aldrich) followed by 24 h incubation in RPMI medium [37].

### 2.4 Cell proliferation assay

The 6 mm Ti disks were placed in 96-well plates. After seeding 1 × 10^3^ cells on Ti-MS and Ti-Al disks for 1, 3, 7, 10, and 14 days, proliferation assays were carried out using the Cell Counting Kit-8 (Dojindo Laboratories, Kumamoto, Japan), in accordance with the manufacturer's instructions. Absorbance was determined using SpectraMax 340PC384 (Molecular Devices, Tokyo, Japan).

### 2.5 Measurement of alkaline phosphatase (ALPase) activity

UE7T-13 cells were plated at a density of 5 × 10^3^ cells on the 6 samples of 15 mm diameter of Ti-MS and Ti-Al. For quantitative analysis of ALPase activity, p-nitrophenol production was measured at 37 °C for 6 min in Milli-Q water using a SIGMAFAST p-Nitrophenyl phosphate Tablet set (Sigma-Aldrich, St. Louis) as a substrate. The relative amount of p-nitrophenol was estimated from the light absorbance at 405 nm on days 7, and 14 using the SpectraMax 340PC384 (Molecular devices).

### 2.6 Alizarin red S staining (ARS)

For staining, 2 × 10^4^ cells were seeded on Ti-MS and Ti-Al (15 mm in diameter) and cultured with DMEM supplemented with 50 μg/mL ascorbic acid (FUJIFILM-WAKO), 100 nM dexamethasone (FUJIFILM-WAKO), and 10 mM β-glycerophosphate (FUJIFILM-WAKO) for 7 and 14 days. Each sample was fixed with 10% formalin and stained with 40 mM ARS solution (FUJIFILM-WAKO) for 30 min at room temperature. After staining, the cells were washed five times with distilled water. The dye was then dissolved using 500 μL of 5% formic acid for 20 min. The extract from 2 M NaCl were diluted into 1 μg/μL with PBS and seeded 30 μL on a 10 mm square Ti-MS and Ti-Al. After incubation, samples were rinsed three times with PBS, and 100 μL of 1 mM CaCl_2_ was added for another overnight incubation at 37 °C 40 mM ARS solution was added to samples for 30 min at 37 °C. the semi-quantification of ARS was as mentioned above.

### 2.7 Immunocytochemical (ICC) analysis

Cells were seeded on Ti disks at a density of 0.5 × 10^2^/cm^2^ and cultured for 24 h. Cells were fixed with 4% paraformaldehyde and permeabilized with 0.1% Triton X-100 in PBS for 10 min. Each disk was stained with DAPI (Vector Laboratories, Inc., Burlingame, CA, USA) and observed under an FV3000 Confocal Laser Scanning Microscope (Olympus, Tokyo, Japan).

### 2.8 TEM (transmission electron microscopy)

5 × 10^2^/cm^2^ cells were seeded on the Ti foil discs of 6 mm diameter. After 7 and 14 days cultivation, samples were washed three times with 0.1 M sodium cacodylate buffer (Nisshin EM Co., Ltd., Tokyo, Japan), fixed with a pre-fixative solution (4% paraformaldehyde (Nisshin EM Co., Ltd), 2% glutaraldehyde (FUJIFILM WAKO), and 0.1 M sodium cacodylate) for 1 h at 4 °C, and immersed in 0.05% ruthenium red solution for 1 h at room temperature A post-fixation was performed with 1% OsO_4_ for 1 h at room temperature, followed by dehydration with ethanol and embedding in EPON resin (Nisshin EM Co., Ltd). Samples were cut with an ultramicrotome (Ultra CUTS, Leica, Tokyo, Japan), stained with lead citrate for 5 s, and observed with a transmission electron microscope (JEM-1400PLUS, JEOL).

### 2.9 Real-time RT-PCR

Cells were seeded on the 32 mm diameter of Ti-MS and Ti-Al at a density of 2 × 10^4^/cm^2^ and cultured for seven and 14 days. Total RNA was isolated using TRIzol LS Reagent (Thermo Fisher Scientific, Waltham, MA, USA), and cDNA was synthesized using ReverTra Ace qPCR RT Master Mix and gDNA Remover (Toyobo Co., Ltd., Osaka, Japan) according to the manufacturer's protocols. Quantitative real-time RT-PCR was performed using THUNDERBIRD SYBR qPCR Mix (Toyobo) and the Mx3000P Real-Time QPCR System (Agilent Technologies International Japan, Ltd., Tokyo, Japan). The expression of target transcripts was normalized against the expression of glyceraldehyde-3-phosphate dehydrogenase *(GAPDH)*, and relative changes in gene expression were determined using the 2^−ΔΔCt^ method.

### 2.10 Immunotolerance assay of hBMSCs, hMps, and T-cells

UE7T-13 cells were cultured on 32 mm Ti-MS and Ti-Al disks for 7 and 14 days. The medium was replaced with DMEM without FBS for 24 h to prepare the conditioned medium (CM). CM was then added to hMps and Jurkat cells and cultured for 24 h. Total RNA was extracted to analyze M1 and M2 macrophage polarization and regulatory T cell (Treg) markers.

### 2.11 Image and statistical analyses

One-way analysis of variance followed by the Student's–Newman–Keuls post-hoc test was used to determine significance. Statistical significance was set at *P < 0.05*.

## 3 Results

### 3.1 Surface characterization of Ti-MS and Ti-Al

**Fig. 1:**
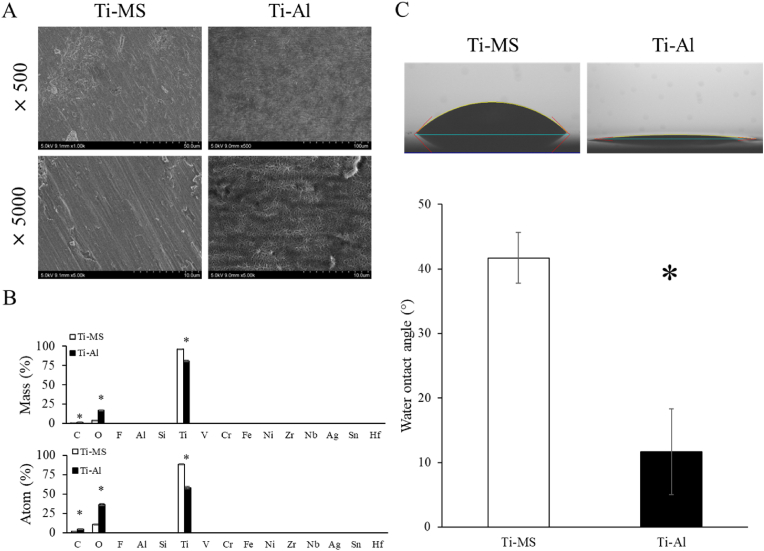
(A) SEM micrograph images of surface of Ti-MS and Ti-Al. (B) Characterization of TiO_2_ surface using WDS analysis. WDS-spectrum quantification of individual elements is shown in Table 1, Table 2. (C) The contact angle on the surface of Ti-MS and Ti-Al. Notes: Data are expressed as means (n = 6) with error bars representing standard deviations; *p < 0.05.

Ti-MS surfaces showed typical machined surface topology (Fig. 1A, left panel). The Ti-Al surface exhibited an alveolate porous structure with homogeneous holes (Fig. 1A, right panel). The results of the elemental analysis of the material surface are shown in Fig. 1B. The atomic and mass percentages of Ti on the surface of Ti-MS were significantly lower than those of Ti-Al. The amounts of C and O on the Ti-Al surface were significantly higher than those on Ti-MS. No other elements were detected in Ti-MS and Ti-Al. Hydrophilicity analysis showed that the Ti-Al surface exhibited more hydrophilic properties than the Ti-MS surface (Fig. 1C).

### 3.2 Cell attachment and proliferation on Ti-MS and Ti-Al

**Fig. 2:**
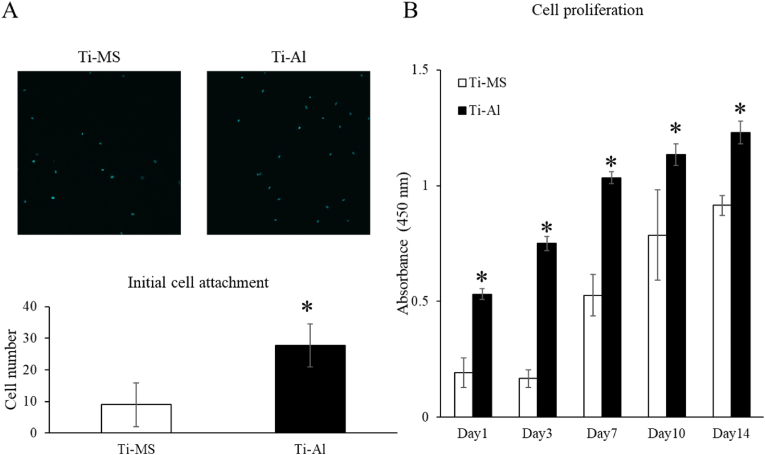
Initial cell adhesion and cell proliferation of UE7T-13 on Ti-MS and Ti-Al. (A) DAPI staining of UE7T-13 cells on Ti-MS and Ti-Al. Bar, 10 μm. (B) Cell proliferation of UE7T-13 on Ti-MS and Ti-Al. Notes: Data are expressed as means (n = 6) with error bars representing standard deviations; *p < 0.05.

Initial cell adhesion of UE7T-13 on Ti-Al was significantly higher than that of UE7T-13 on Ti-MS (Fig. 2A). At all time points, the number of cells on Ti-Al was significantly higher than that on Ti-MS (Fig. 2B).

**Table 1:** Weight % and Atomic % of components on Ti-MS and Ti-Al disks by SEM-WDS.

Gene name	Forward primer	Reverse primer	Accession#
GAPDH	AGCAAGAGCACAAGAGGAAGAG	TCTACATGGCAACTGTGAGGAG	NM_001289745.2
Runx2	AAGCTTGATGACTCTAAACC	TCTGTAATCTGACTCTGTCC	NM_001024630.3
ALP	AGCTGAACAGGAACAACGTG	ATTCTGCCTCCTTCCACCAG	NM_000478.5
Col-Ia	GCTATGATGAGAAATCAACCG	TCATCTCCATTCTTTCCAGG	NM_000088.3
OCN	AATCCGGACTGTGACGAGTTG	CTGGAGAGGAGCAGAACTGG	NM_199173.6
IDO	TCCTTACTGCCAACTCTCCAAG	CGTCCATGTTCTCATAAGTCAGG	AH002828.2
IL-6	AATGAGGAGACTTGCCTGGTG	TGTACTCATCTGCACAGCTCTG	NM_001318095.1
IL-10	ATCAAGGCGCATGTGAACTC	AAGGCATTCTTCACCTGCTC	NM_000572.3
TSG-6	CCAGGCTTCCCAAATGAGTA	AAAGCCATGGACATCATCGT	NM_007115.4
CD4	TCGCGCTGACTCAAGAAGAA	TTGGCAGTCAATCCGAACAC	MK170450.1
CD25	CCTGGGACAACCAATGTCAA	TTTTCCCATGGTGGAGGTTC	NM_000417.3
CD80	GTTATCCACGTGACCAAGGAAG	TTGTGCCAGCTCTTCAACAG	NM_005191.4
CD86	ACTGTACGACGTTTCCATCAGC	AGCCGCGTCTTGTCAGTTTC	KU284848.1
CD163	AAGACGCTGCAGTGAATTGC	AATGGCCAACAGAACAACCC	DQ058615.1
CD206	TGGAGCAGGTGGAAGATCTATG	ACTTGAACGGGAATGCACAG	NM_002438.4
FoxP3	GCCATGGAAACAGCACATTC	AGGCAAACATGCGTGTGAAC	EF534714.1
FAM20B	GCTGTTGAGCACCTTCCTAAC	ATGTCTCCATCAGCACAAGC	NM_014864.3
XylT-1	AAGTGCGAACAGACAGCAAC	TGTCTACTCGGTGGCTTCTTC	NM_022166.3
XylT-2	TTCAAGCCACAGGACTTCCTC	TGGTTCACAGTCGACTCGAAC	NM_022167.3
CHST11	CATCAGGTTGGTGTGATGCAG	ACCATGCACAGCACACATTG	NM_001173982.1
CHST12	TCGGTGTTCATGATCCTGCTG	AGAGAAGGACGTGTGCAAGTAG	NM_001243794.1
D4ST-1	ATCCGAGAGTACCAGCAACG	ATCTCAGGAACTCGGGGAAT	NM_130468.3
C6ST-1	AAGCAGATTCCCCAAGCTCT	GGGACAAGAGAGATGCGTTC	NM_004273.4

Abbreviations: GAPDH; glyceraldehyde-3-phosphate dehydrogenase, Runx2; Runt-related transcription factor 2, ALP; Alkaline phosphatase, Col-Ia; Collagen Type I, OCN; Osteocalcin, IDO; Indoleamine-pyrrole 2,3-dioxygenase, IL; Interleukin, CD; Cluster of differentiation, FoxP3Forkhead box P3, FAM; Family with sequence similarity member, XylT; Xylosyltransfwrase, CHST; Carbohydrate sulfotransferase, D4ST; Dermatan 4-0 sulfotransferase, C6ST; Chondroitin 6 sulfotransferase.

**Table 2:** Elemental composition of samples.

	Mass percentage		Atomic percentage	
	Ti-MS	Ti-Al	Ti-MS	Ti-Al
C	0.43 ± 0.02	1.61 ± 0.21	1.60 ± 0.09	4.60 ± 0.54
O	3.77 ± 0.24	16.9 ± 0.89	10.3 ± 0.62	36.4 ± 1.32
F	0 ± 0	0 ± 0	0 ± 0	0 ± 0
Al	0 ± 0	0 ± 0	0 ± 0	0 ± 0
Si	0 ± 0	0 ± 0	0 ± 0	0 ± 0
Ti	95.7 ± 0.26	80.7 ± 0.99	88.0 ± 0.67	57.9 ± 1.66
V	0 ± 0	0 ± 0	0 ± 0	0 ± 0
Cr	0 ± 0	0 ± 0	0 ± 0	0 ± 0
Fe	0 ± 0	0 ± 0	0 ± 0	0 ± 0
Ni	0 ± 0	0 ± 0	0 ± 0	0 ± 0
Zr	0 ± 0	0 ± 0	0 ± 0	0 ± 0
Nb	0 ± 0	0 ± 0	0 ± 0	0 ± 0
Ag	0 ± 0	0 ± 0	0 ± 0	0 ± 0
Sn	0 ± 0	0 ± 0	0 ± 0	0 ± 0
Hf	0 ± 0	0 ± 0	0 ± 0	0 ± 0

### 3.3 Osteogenic activity of UE7T-13 on Ti-MS and Ti-Al

**Fig. 3:**
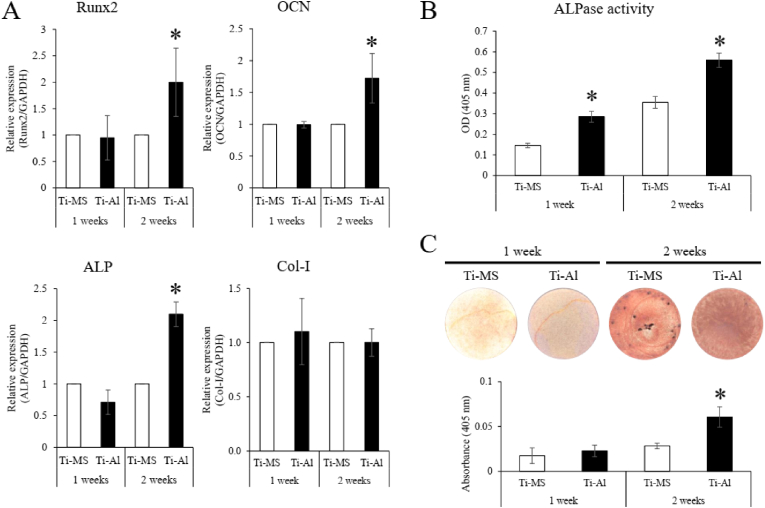
Osteogenic capacity of UE7T-13 on Ti-MS and Ti-Al. (A) Gene expression of Runx2, OCN, ALP, and Col-I in UE7T-13 on Ti-MS and Ti-Al. (B) ALP activity in UE7T-13 on Ti-MS and Ti-Al. (C) UE7T-13 were stained after 1- and 2- week cultivation. The staining intensity is quantified in the graph. Notes: Data are expressed as means (n = 6) with error bars representing standard deviations; *p < 0.05.

Runt-related transcription factor-2 (Runx2), is known as a regulator of bone development. Runx2 controls osteocalcin (OCN) and alkaline phosphatase (ALP) expression which play important roles in mineralization. Type I collagen (Col-I) gene and its product is the main organic component in bone and serves as a template for mineralization [38]. These genes are markers of BMSC differentiation from osteoprogenitors to osteoblasts. Runx2, OCN, and ALP expression in UE7T-13 on Ti-Al was significantly higher than that on Ti-MS after two weeks of cultivation; however, no significant differences were observed after one week of cultivation. Furthermore, there were no significant differences in Col-I expression between the UE7T-13 cells cultured on Ti-MS and Ti-Al (Fig. 3A). The ALPase activity in UE7T-13 on Ti-Al was significantly higher than that in UE7T-13 on Ti-MS at 1 and 2 weeks (Fig. 3B). The number of calcium nodules stained with ARS in UE7T-13 on Ti-Al was significantly higher than that in UE7T-13 on Ti-MS after 2 weeks of cultivation (Fig. 3C).

### 3.4 Expression of glycosyltransferase in hBMSCs on Ti-MS and Ti-Al

**Fig. 4:**
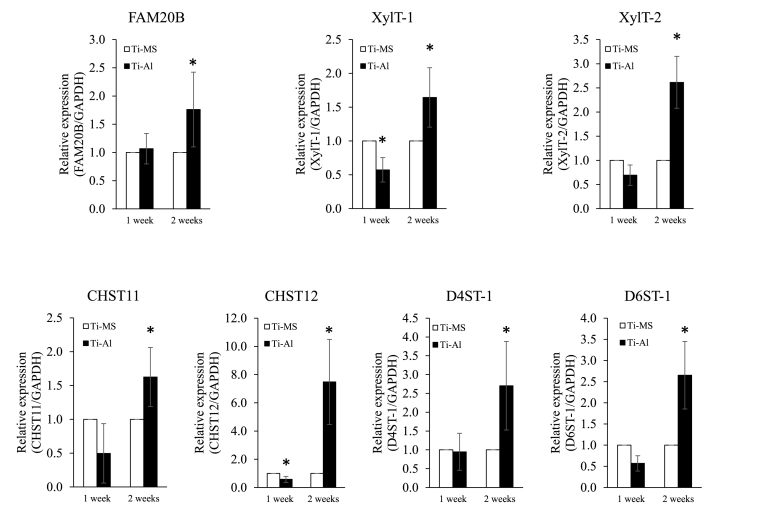
Gene expression of glycosyltransferases in UE7T-13 cultured on Ti-MS and Ti-Al at 1- and 2-weeks cultivation. Notes: Data are expressed as means (n = 3) with error bars representing standard deviations; *p < 0.05.

After 1 week of cultivation, the expression of XylT-1 in UE7T-13 on Ti-Al was significantly lower than that in UE7T-13 on Ti-MS. After two weeks of cultivation, the expression of all glycosyltransferases in UE7T-13 on Ti-Al was significantly higher than that in UE7T-13 on Ti-MS (Fig. 4).

### 3.5 Ultrastructural analysis of the PG-layer

**Fig. 5:**
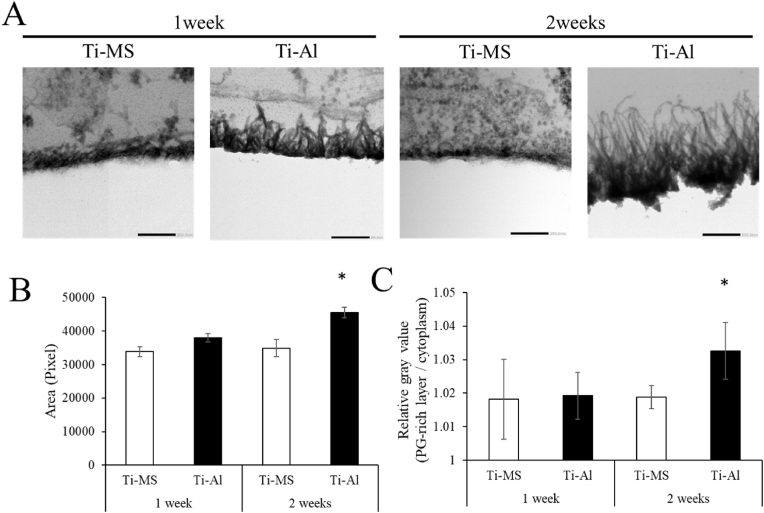
PG-layer formation *in vitro*. (A) TEM images between Ti-MS and -Al surface and UE7T-13 at 1- and 2-week cultivation. (B) pixel or (C) relative gray value (PG-rich layer/cytoplasm). The bar represents 500 nm (upper) and 100 nm (lower), respectively. Three samples were prepared each experimental group, and fifteen images in total were analyzed. *p < 0.05 vs. UE7T-13 cultured on Ti-MS.

At 1 and 2 weeks, PGs formed on Ti-Al had needle-like structures extending across the UE7T-13 interface. However, the PGs formed on Ti-MS did not exhibit a spike-like shape or vague staining between UE7T-13 and Ti-MS (Fig. 5A). The area and relative gray value of the PG-layer formed on Ti-Al were significantly higher than those formed on Ti-MS after 2 weeks of cultivation (Fig. 5B).

### 3.6 Immunotolerance capacity of UE7T-13 on Ti-MS and Ti-Al

**Fig. 6:**
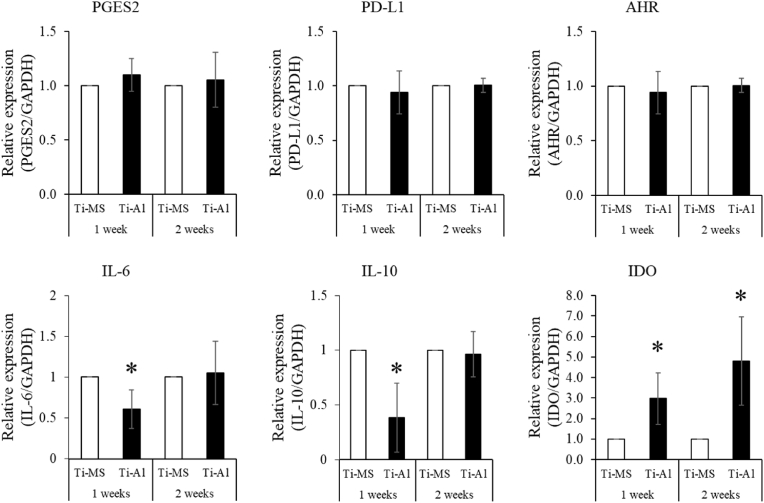
Immunotolerance-related gene expression in UE7T-13 on Ti-MS and Ti-Al. (A) Gene expressions of PGES2, PD-L1, AHR, IL-6, IL-10, and IDO in UE7T-13 on Ti-MS and Ti-Al. Notes: Data are expressed as means (n = 6) with error bars representing standard deviations; *p < 0.05.

The gene expressions of PGES2, PD-L1, and AHR were not significantly different in UE7T-13 between Ti-MS and Ti-Al at any time point. The expressions of IL-6 and IL-10 in UE7T-13 on Ti-Al were significantly lower than those in UE7T-13 on Ti-MS after one week of cultivation; however, the difference was not significant after two weeks of cultivation. The gene expression of IDO in UE7T-13 cells on Ti-Al was significantly higher than that in UE7T-13 cells on Ti-MS at all time points (Fig. 6).

### 3.7 Immunotolerance capacity of hMps and T-cell affected by UE7T-13 on Ti-MS and Ti-Al

**Fig. 7:**
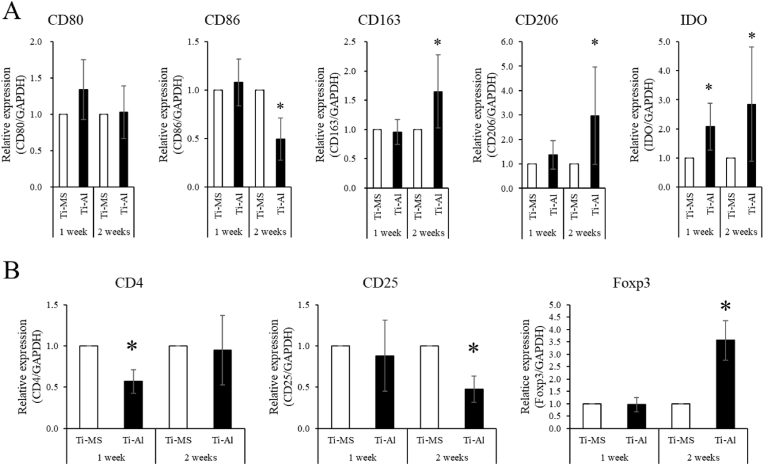
Effect of indirect immunotolerance in UE7T-13 cultured on Ti-MS and Ti-Al. (A) Gene expression related to M1-and M2- Mps marker in CM-treated hMps derived from UE7T-13 on Ti-MS and Ti-Al at 1- and 2-week cultivation. (B) Gene expression of Treg marker in CM-treated Jurkat derived from UE7T-13 on Ti-MS and Ti-Al. Nine analyses were performed in total. *p < 0.05 vs. Ti-MS. The figures are representative of three independent experiments; six analyses were performed in total. *p < 0.05.

Gene expressions of CD86, CD163, CD206, and IDO in hMps treated with CM derived from UE7T-13 cultured on Ti-Al were significantly different from those in hMps treated with CM derived from UE7T-13 cultured on Ti-MS (Fig. 7A). In contrast, the gene expression of CD4 in T-cell treated with CM derived from UE7T-13 on Ti-Al was significantly lower than that on Ti-MS after 1 week of cultivation. The expression of CD25 in T-cell treated with CM derived from UE7T-13 on Ti-Al was significantly lower than that on Ti-MS after 2 weeks of cultivation. The gene expression of Foxp3 in T-cell treated with CM derived from UE7T-13 on Ti-Al was significantly higher than that on Ti-MS after 2 weeks of cultivation (Fig. 7B).

### 3.8 Discussion

In this study, we analyzed whether alkali-treated TiO_2_ affected the immunotolerance and osteogenic capacity in BMSCs. We first characterized the surface of Ti-MS and Ti-Al, and the results were similar to those found in a previous study [9]. These results suggested that treatment of ultrasonication was useful, because the treatment time could be shortened.

The results of the initial cell attachment and cell proliferation also corresponded to those of previous studies. In contrast, the expression of osteogenesis-related genes exhibited some differences [18,19]. In previous reports, the expression of Col-I in BMSCs cultured on Ti-Al was significantly higher than that in BMSCs cultured on Ti-MS. This difference was suggested to be caused by differences in the cells. In contrast, the calcification capacity of the hBMSCs on Ti-Al was consistent with previous reports. The alkali treatment of TiO_2_ in this study promoted osteogenic properties compared with non-treated TiO_2_.

We investigated the relationship among the expression of glycosyltransferases, calcification capacity, and initial cell attachment in hBMSCs cultured on Ti-MS and Ti-Al. FAM20B is a glycan kinase essential for the morphogenesis and mineralization of the craniofacial complex [39,40]. Increased levels of XylT-1 correlate with mineralization of the extracellular matrix during osteogenic differentiation of mesenchymal stem cells [41]. Highly sulfated CS promotes osteogenic differentiation because the 4,6-disulfates in N-acetyl-galactosamine provide binding sites for these protein ligands [30,42]. Enzymatic digestion of CS impairs the formation of mineral modules [43]. CS and DS play important roles in cells involved in osteoblast-like cell adhesion [44]. The results of the present study are consistent with those of previous studies. FAM20B, XylT-1, and CS have been suggested to play important roles in calcification and cell proliferation on TiO_2_.

Ultrastructural images suggested that a PG-layer formed along the fine protrusions of Ti-MS and Ti-Al as a template, and surface roughness on Ti-Al was suggested to contribute to the promotion of the area of the PG-layer. Needle-like crystals composed of mineral substances and/or calcified nodules have been observed in collagen fibers in bone tissue [45,46]. Furthermore, these results correspond to those identified using ARS. It has been suggested that the calcified nodules on Ti-Al are more similar to those on natural bone. Thus, the surface modification of TiO_2_ changed the morphology, area, and function of the PG-layer.

The chromatograms of PGs eluted from hBMSCs on Ti-MS and Ti-Al corresponded to a previous study and were shown to extract proteoglycan in a 2.0 M NaCl fraction [47]. However, these results did not correspond to ultrastructural observations because absorbance at 280 nm only detected amino acids and not glycan. The smeared bands of PGs in hBMSCs cultured on Ti-Al did not shift the low molecular weight in Stains-All staining. This suggests that the effects of the surface topology and wettability of Ti-Al on GAG quantity in the PG-layer were relatively small. Furthermore, the calcification capacity results suggested that PGs derived from BMSCs cultured on Ti-Al had the ability to improve mineralization compared to those cultured on Ti-MS. Previous reports have shown that the modification of GAG components in biomaterials changes osteogenic activity in the field of bone regeneration [48]. However, the detailed components of the GAGs containing PGs on Ti-MS and Ti-Al were not revealed in this study. Future studies should investigate the GAG components of individual PGs, such as decorin and biglycan.

During the healing phase of tissue repair and immune-mediated diseases, mesenchymal stem cells, including BMSCs, control immune cells such as T cells, macrophages, and dendritic cells [49,50]. We previously reported that C4S, a GAG, affects the immunotolerance of BMSCs on TiO_2_ [3]. Alkali treatment of TiO_2_ promoted the polarization of macrophage-like cell lines into M2 macrophages [22,51,52]. Our results suggest that BMSCs on Ti-Al directly promote immunotolerance. BMSC-derived IL-6 and PGE2 stimulate M2 macrophage differentiation and IL-10 secretion from macrophages, thus reducing inflammation [53,54]. Moreover, BMSC-derived IL-10 generated M2 macrophages, which create an immunosuppressive microenvironment [49]. In our results, hMps tended to differentiate into M2 macrophages, but did not differentiate completely because CD80 expression was not significantly different between the two experimental groups. Our results suggest that the difference in IL-6 and IL-10 expression between the two experimental groups was too small to differentiate macrophage polarization, because differences in gene expression were approximately 2- to 3-fold. IDO is the rate-limiting enzyme in the tryptophan catabolic pathway and is regarded as one of the key modulators of acquired immune tolerance [55]. These results suggest that the PG-layer controls immunotolerance around TiO_2_ directly and indirectly. Furthermore, IDO affects the generation and expansion of Tregs [56]. Tregs are identified by the expression of CD4, CD25, and Foxp3 [57,58]. However, the expression of the Treg marker in Jurkat cells treated with CM derived from hBMSCs cultured on Ti-Al was atypical. CD25 and Foxp3 expression was very low because the cycle threshold of CD25 and Foxp3 in Jurkat cells ranged from 30 to 33 (data not shown). The cycle threshold of Foxp3 in Jurkat cells treated with DMEM without FBS was in a similar range, 31–33 (Supplemental data.1). On the other hand, Foxp3 expression in Jurkat cells is originally higher than that in other cancer cells [59]. From the above, it was suggested that low expression of Foxp3 in this study was caused by the lack of FBS in CM. Stronger stimulation, such as co-culture system [60], gene transduction [61], IDO inhibitor [62], and suppression assays [63] will be needed for Jurkat cells to differentiate into Tregs. In the future, we should investigate the proteins and micro-RNA in CM to determine the factors that induce the differentiation of Jurkat cells into Tregs. Furthermore, we will investigate the function of identified factors in BSMCs on Ti-MS and Ti-Al to immune cells using gene silencing methods, such as small hairpin RNA.

## 4 Conclusion

This study revealed that alkali-treated TiO_2_ promotes the gene expression of glycosyltransferase in hBMSCs and alters the morphology and quantity of the PG-layer that was formed by hBMSCs. Alkali -treatment of TiO_2_ promoted the calcification capacity of PGs derived from hBMSCs and affected their immunotolerance capacity of hBMSCs both directly and indirectly *in vitro*.

## Funding

This work was supported in part by grants from the JAPAN SOCIETY FOR THE PROMOTION OF SCIENCE [Kakenhi Kiban C, 21K09955, to Shuhei Tsuchiya] and the TERUMO LIFE SCIENCE FOUNDATION.

## Author contributions

Shuhei Tsuchiya and Tomomi Mizutani contributed to all the experiments, including cell culture, titanium materials, and gene and protein expression analyses. Masaki Honda contributed to the analysis of the titanium surfaces. Kensuke Kuroda contributed to the preparation of Ti samples. Yasuyuki Shibuya, Jorge Luis Montenegro Raudales, Hironori Miyamoto, Tomohisa Nakamura, and Kenichiro Ishibashi revised the manuscript and provided suggestions for the study.

## Declaration of competing interest

The authors declare that they have no known competing financial interests or personal relationships that could have influenced the work reported in this paper.
