# Supplementary figures and images for: Alkali-treated titanium dioxide promotes formation of proteoglycan layer and altered calcification and immunotolerance capacity in bone marrow stem cell
Source: Biochem Biophys Rep. 2023 Nov 9;36:101569. doi: 10.1016/j.bbrep.2023.101569 (PMC10658208; doi:10.1016/j.bbrep.2023.101569)

**List of primers**


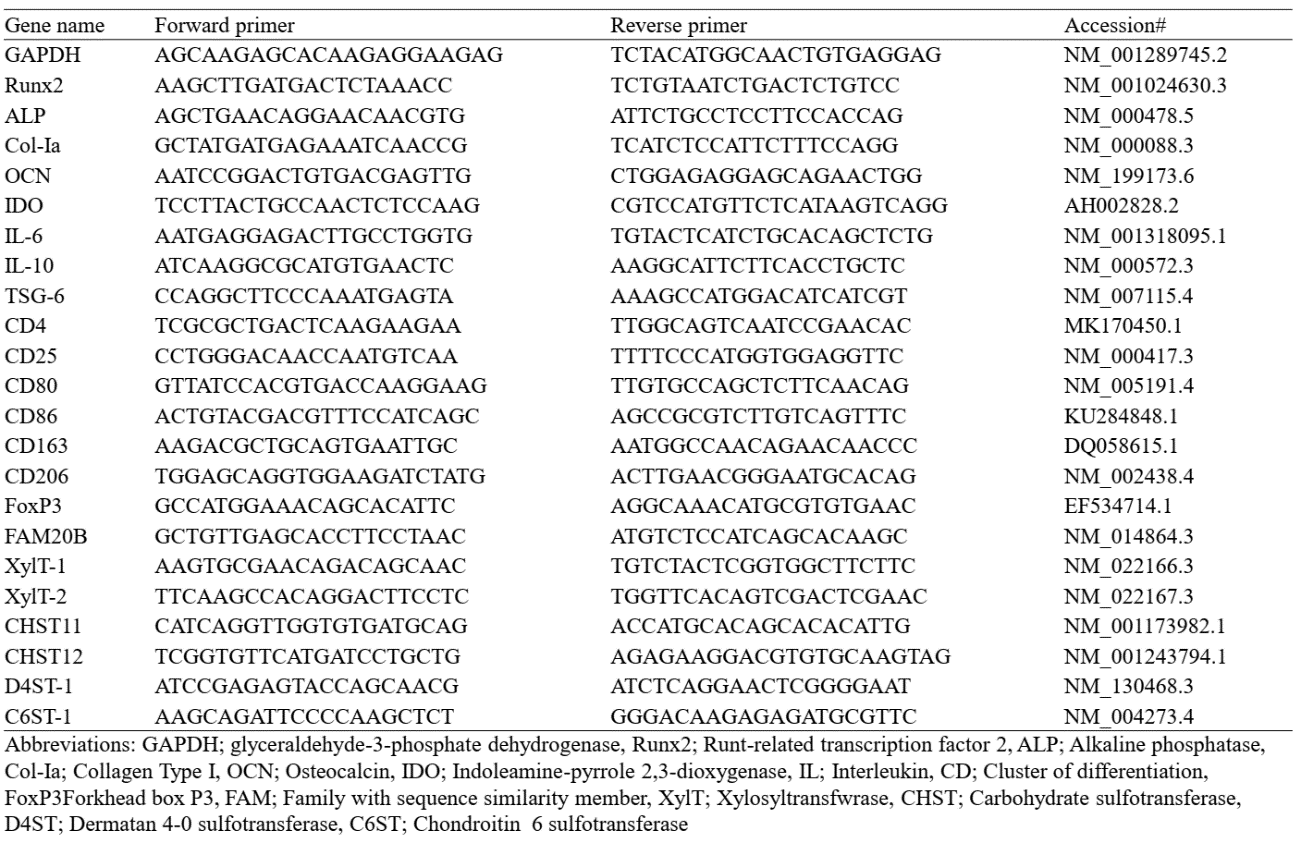


**Supplemental data. 1**


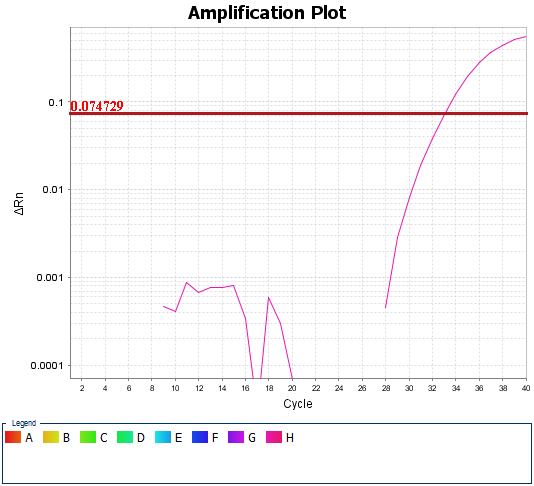


Cycle threshold of Jurkat cell in DMEM without FBS.

Supplement: Multimedia component 1 [file mmc1.docx]
